# Early Perturbations in Glucose Utilization in Malaria-Infected Murine Erythrocytes, Liver and Brain Observed by Metabolomics

**DOI:** 10.3390/metabo10070277

**Published:** 2020-07-07

**Authors:** Arjun Sengupta, Soumita Ghosh, Shobhona Sharma, Haripalsingh M. Sonawat

**Affiliations:** 1 Department of Chemical Sciences, Tata Institute of Fundamental Research, Homi Bhabha Road, Mumbai 400005, India; soumita@upenn.edu (S.G.); harisonawat@gmail.com (H.M.S.); 2 Department of Biological Sciences, Tata Institute of Fundamental Research, Homi Bhabha Road, Mumbai 400005, India; shobhona@gmail.com

**Keywords:** malaria, glucose utilization, ^13^C NMR, Balb/c, *Plasmodium berghei* ANKA, metabolism

## Abstract

Investigation of glucose utilization during an infection is central to the study of energy metabolism. The heavy utilization of glucose by the malaria parasite, and the consequences of this process, have been investigated extensively. However, host glucose utilization during early infection has not been explored to date. In a first attempt, this article investigates the changes in the host glucose utilization in Balb/c mice infected with *Plasmodium berghei* ANKA using ^13^C-labeled glucose infusion followed by NMR spectroscopy. The results suggested significant alterations of liver, brain and red blood cell (RBC) glucose utilization during early infection when the parasitemia was <1%. At the pathway level, we observed a decrease in the shunt metabolite 2,3-bisphosphoglycerate in the RBCs. Glycolysis and pathways associated with it, along with fatty acid unsaturation, were altered in the liver. Significant changes were observed in the central carbon metabolic pathways in the brain. These results have implications in understanding the host physiology during early infection and pave the way for detailed flux analysis of the proposed perturbed pathways.

## 1. Introduction

Alteration of the metabolic network during severe infection by malarial parasites has long been known. This has been attributed to the different tissues affected by the parasite [1,2]. In severe cases of *Plasmodium falciparum* infection, the parasitized red blood cells exhibit sequestration in the blood vessels. This, in turn, affects the microvasculature of several tissue types, perturbing the physiology of tissues/organs such as the liver, heart, kidney, lungs, adipose tissue, retina, etc. [3,4,5]. This is expected to generate localized metabolic stress. As the disease approaches its late stage, the inflammatory immune responses generated by the infection lead to severe complications such as liver damage, renal malfunction, cerebral malaria, hypoglycemia, and acidosis, which are often causes of death [6,7,8]. Hypoglycemia and acidosis are often reported as the major metabolic complications of severe malaria. Hypoglycemia during severe malaria has been attributed to several factors, such as cytokine-induced impairment of gluconeogenesis, starvation and malabsorption of glucose, increased tissue metabolism, and excessive utilization of glucose by intraerythrocytic parasites [9]. In addition, acidosis, especially due to accumulation of lactic acid, is an important factor and an indicator of death associated with the infection. The pathophysiology of acidosis remains confusing [10], however, elevated glucose utilization through anaerobic glycolysis by the parasites is a possible reason [11]. Glycolysis is known to be the only glucose utilization and ATP generation pathway in the asexual stages of the *Plasmodium* sp. parasites for which the parasite channels the host glucose sources [12]. Glycolysis is known to be up-regulated in heavy loads of parasitemia in a murine model of infection [13] as well as in an in vitro culture of *P. falciparum* [14]. Therefore, an important question that remains to be asked is how the host utilizes its glucose resources in different tissues and cells. Especially, the early stages of infection should be of importance, because the host–parasite interaction biology is still not over-burdened by the parasite at this point. Earlier studies have shown altered gluconeogenesis in the liver of cerebral malaria patients [15]. However, little to no data exist about glucose utilization by the host during the early stage of infection.

Here, an attempt has been made to delineate the perturbation of the cellular/organ level glucose utilization network in a murine model at the early stage of malaria infection. The altered glucose metabolism network has been evaluated in the liver, brain, and red blood cells (RBCs) of infected female mice with respect to uninfected mice when the parasitemia level of the infected mice was ~1% (indicative of the early stage of infection). Specifically, we investigated the changes in labeled metabolite pools in infected and uninfected animals. This was achieved by ^13^C NMR/^1^H-detected ^13^C HSQC NMR spectroscopy and a targeted metabolomics approach.

## 2. Results

### 2.1. NMR Spectroscopy of Organ and Cellular Extracts

**Figure 1:**
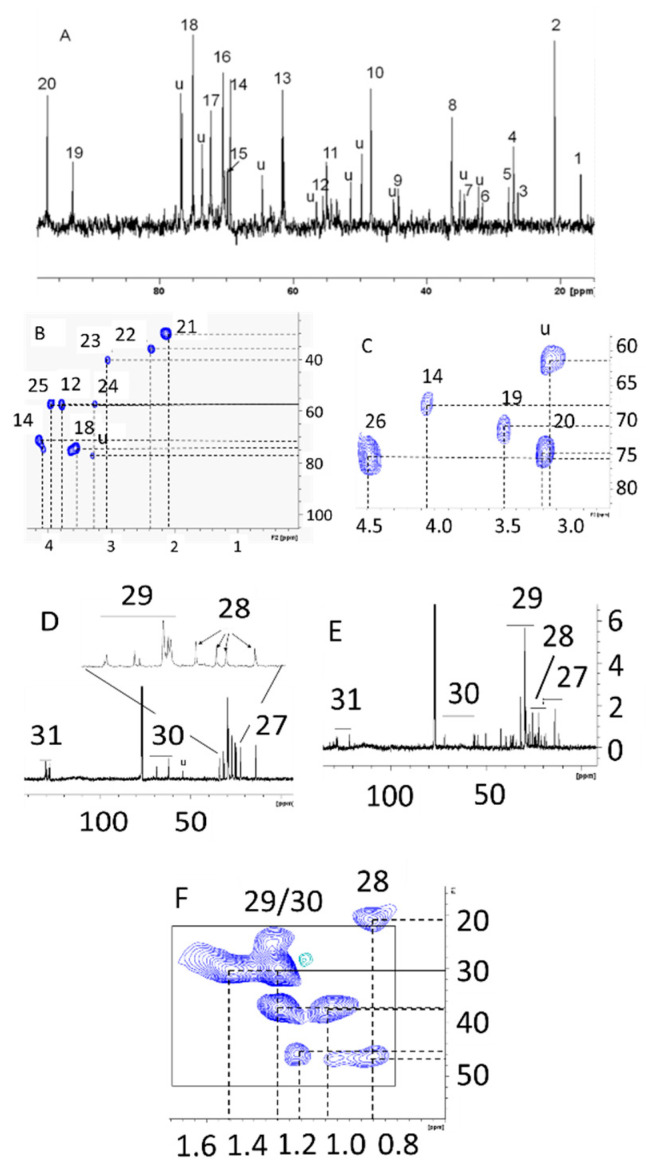
Representative NMR spectral profile from uninfected healthy control Balb/c mouse after infusion of (2^13^C)glucose through tail vein. (**A**) 1-dimensional ^13^C NMR profile of hydrophilic extract of liver; (**B**,**C**) 2-dimensional HSQC profiles of hydrophilic extracts of red blood cells (RBCs) and brain, respectively; (**D**,**E**) 1-dimensional ^13^C NMR profile of hydrophobic extracts of liver and brain, respectively; (**F**) 2-dimensional HSQC profile of hydrophobic extract of RBC. Keys: 1-C3-alanine, 2-C2-lactate, 3-C3-N-acetylcysteine, 4-C3-glutamine, 5-C2-acetate, 6-C4-glutamine, 7-unidentified, 8-C2-taurine, 9-guanidoacetate, 10-C1-taurine, 11-Choline (N-CH_3_), 12-C2-alanine, 13-C6-glucose (α + β), 14-C2-lactate, 15-C2-cysteine, 16-C2-glycerol, 17-C2-α-glucose, 18-C2-β-glucose, 19-C1-α-glucose, 20-C1-β-glucose, 21-C3-glutamate, 22-C4-glutamate, 23-Creatine (N-CH_3_), 24-betaine (N-CH_3_), 25-C2-Creatine, 26-C2-2,3-BPG,27-cholesterol CH_3_, 28-cholesterol/fatty acid CH2, 29-fatty acid/TG CH_2_, 30-long chain TG Glycerol CH_2_, 31-long chain fatty acid unsaturated carbon.

Malarial infection was shown to heavily alter peripheral and tissue level metabolite levels [16,17,18,19,20,21,22,23,24,25,26] These studies are carried out after the infections are well established, but alterations in rodent host metabolism during low parasitemia (<1%) are unknown. In RBC cultures at ~5% parasitemia, significant alterations in glucose utilization have been observed [14,27], and thus it was of interest to study whether glucose utilization will be affected in the RBCs, liver, and brain of the host during this early stage of malarial infection. To probe this, NMR spectroscopy was performed on the hydrophobic and hydrophilic extracts of the liver, brain, and RBCs to assess the labeled metabolite pool after 30 min of injection of the labeled glucose. We performed 1-dimensional ^13^C NMR spectroscopy in the liver samples and 2-dimensional ^1^H-^13^C HSQC spectroscopy on the RBC and brain extracts. The choice of 1-dimensional or 2-dimensional spectroscopy was solely dependent upon the number of metabolites detected in each sample type. Specifically, 1-dimensional experiments didn’t provide an appreciable number of signals from RBC and brain extracts. For this reason, we acquired more sensitive ^1^H-detected 2-dimensional experiments for those sample types. The representative NMR spectra and the metabolite assignments of the specific organs and cellular extracts (both hydrophilic and hydrophobic) of samples from infected mice are shown in Figure 1.

### 2.2. Alteration in Glucose Utilization of RBC

**Figure 2:**
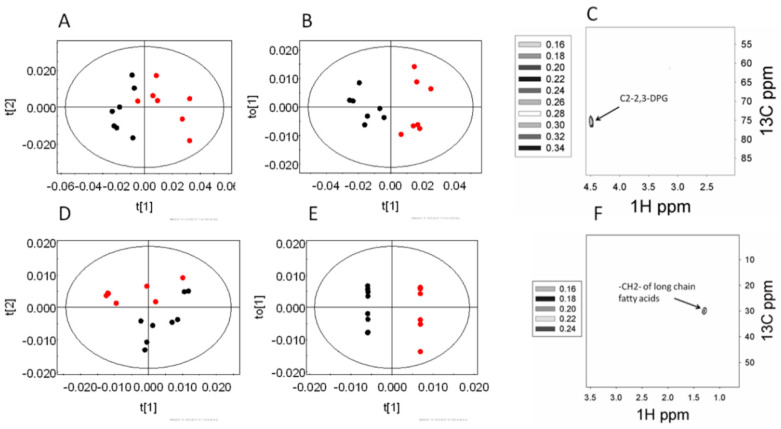
The changes in the labeling patterns from (2^13^C)glucose in the RBCs of early stage infection (~1% parasitemia) and uninfected control mice. (**A**–**C**) hydrophilic extract, and (**D**–**F**) hydrophobic extract of the RBC. (**A**,**D**) Principal component analysis (PCA) score plot and (**B**,**E**) orthogonal partial least square discriminant analysis (OPLSDA) score plots of the corresponding models; black—early stage, and red—control animals. (**C**,**F**) Relevant OPLSDA loadings plot. The contour levels indicate the OPLSDA loadings.

Previously, our group has shown that ~5% of *P. falciparum*-infected RBCs significantly impact glucose utilization from uninfected RBCs from the same culture [14]. In *P. falciparum* infections, the parasitemia in patients typically remains far less than 5%, whereas in the rodent model of *Plasmodium berghei* infection the parasitemia reaches 20% to 40%. Therefore, ~1% infection in this rodent model does indicate a very early stage, and it was interesting to note that the labeling pattern in the RBC was distinct even when the mice had ~1% parasitemia, compared to that of the uninfected control animals. Principal component analysis (PCA) of the hydrophilic fraction of RBCs showed variation along the first principal component (PC1 R^2^X = 0.48) as did orthogonal partial least square discriminant analysis (OPLSDA) modeling (Q^2^(cum) = 0.55). This is evident from the score plots (Figure 2A,B). The extent of labeling in the (2-^13^C)2,3-bisphosphoglycerate was found to decrease in the infected animals (Figure 2C). On the other hand, the hydrophobic fraction of RBCs showed significant variations, which were captured along the PC2 (R^2^X = 0.23 of the PCA scores) (Figure 2D). As expected, the OPLSDA score plot (Figure 2E) also showed significant distinction among the two classes (Q^2^(cum) = 0.82). The loadings plot indicated a decrease in the labels in the long chain fatty acids (Figure 2F).

### 2.3. Alteration in Glucose Utilization of Liver

**Figure 3:**
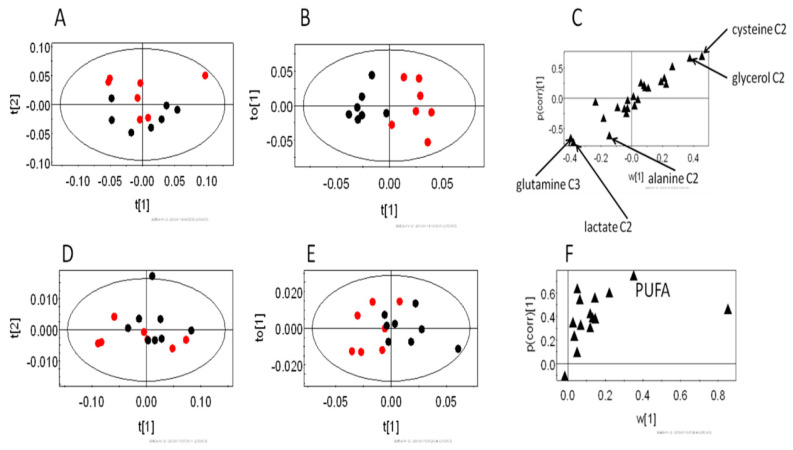
The changes in the labeling patterns from (2^13^C) glucose in the livers of early stage infection (~1% parasitemia) and uninfected control mice. (**A**–**C**) hydrophilic extract and (**D**–**F**) hydrophobic extract of the liver. (**A**,**C**) PCA scores plot and (**B**,**E**) OPLSDA score plots of the corresponding models; black—early stage, and red—control animals. (**C**,**F**) relevant OPLSDA loadings plots. PUFA: polyunsaturated fatty acids. The assignments of significant loadings are tabulated in Supplementary Table S1.

The liver of an animal handles the toxic elements of an infection. Since RBCs showed an effect on glucose metabolism at ~1% parasitemia, we hypothesized that the liver tissue would also show alterations in the glucose utilization in infected versus uninfected mice. Analysis of the hydrophilic fraction of the liver extracts showed a distinct labeling pattern in the multivariate data analysis. The PCA scores (Figure 3A) showed certain distinction across the PC2 (R^2^X = 0.19). The OPLSDA score plot (Figure 3B) also indicated clear segregation (Q^2^(cum) = 0.51). The OPLSDA loadings plot (Figure 3C) was further analyzed. This revealed that (2-^13^C) cysteine and (2-^13^C) glycerol labeling was lower in the infected animals. However, the labeling of each of (2-^13^C) alanine, (2-^13^C) lactate and (3-^13^C) glutamine was higher in the infected animals. Further analysis on the hydrophobic fraction of the liver extracts revealed almost no segregation in the PCA scores plot (Figure 3C). However, OPLSDA scores plot revealed certain distinction among the infected and the uninfected control animals (Figure 3D). From the loadings plot, an elevation in the labeling of unsaturated carbons of the polyunsaturated fatty acids (PUFA) was observed.

### 2.4. Alteration in Glucose Utilization of Brain

Our group has previously shown significant changes in global cerebral metabolite levels in both cerebral and non-cerebral malarial infection in rodents [16,17,18,19,20,21,22,23,24,25,26]. Due to the blood–brain barrier, infected RBCs do not gain access to the brain. However, the brain is a heavy user of systemic glucose, and hence it was of interest to note whether the cerebral glucose utilization was also impacted in the host at such an early stage of infection.

**Figure 4:**
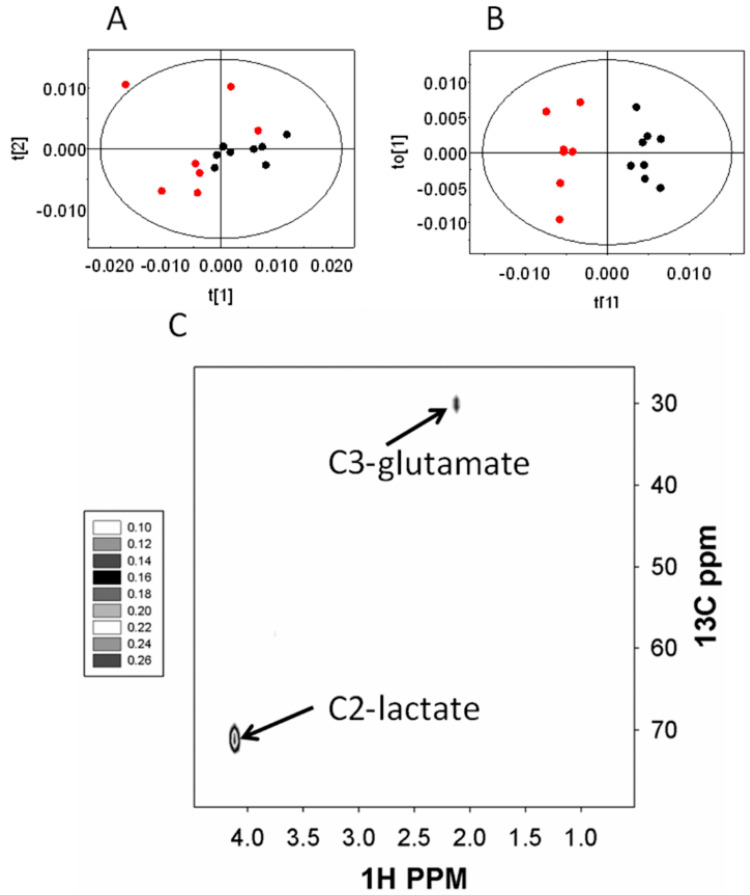
The changes in the labeling patterns from 2^13^C-glucose in the brain of early stage infection (~1% parasitemia) and uninfected control mice. (**A**–**C**) hydrophilic extract brain. (**A**) PCA score plot and (**B**) OPLSDA score plot; black—early stage, and red—control animals. (**C**) relevant OPLSDA loadings plot.

**Figure 5:**
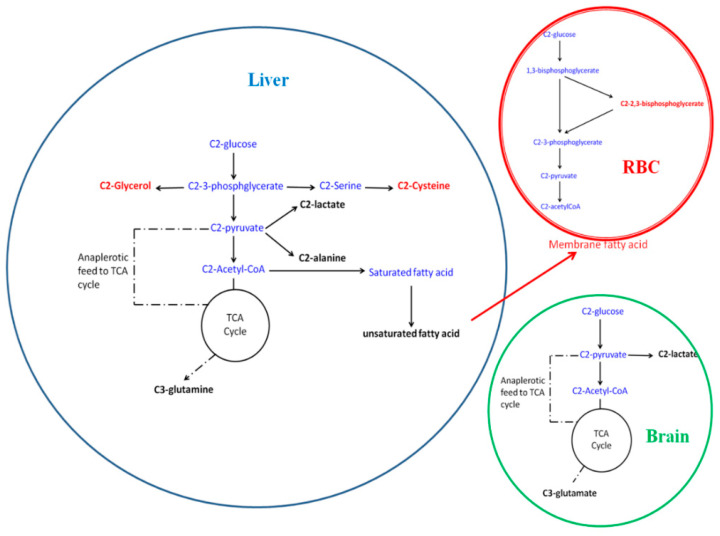
The altered labeling pattern of glucose during early stage malarial infection. The red labels are decreased during the infection, while the black labels are increased. Blue labels are not altered or not picked up in the analysis. The red arrow indicates decreased transport phenomena.

In the hydrophilic fraction of the brain, significant distinction was found between the infected mice and the uninfected control animals. In the PCA scores plot (Figure 4A) this was evident along the PC1 itself (R^2^X = 0.28). The OPLSDA score (Figure 4B) also showed significant differences among these two classes (Q^2^(cum) = 0.30). The OPLSDA loadings plot revealed that the difference came from elevated labeling of (2-^13^C)lactate and (3-^13^C)glutamate (Figure 4C). The hydrophobic fraction of the brain, however, did not show any significant alteration in the labeling patterns of metabolites during the early stage of the infection. A summary of the glucose utilization observations is shown in Figure 5.

## 3. Discussion

Metabolism of glucose is one of the most crucial processes to maintain the energy balance of an organism. Under normal circumstances, blood glucose level is tightly regulated through homeostatic maintenance. This is achieved by the intricately connected network of glycolysis-gluconeogenesis and glycogenolysis-glycogenesis. Alteration in any of these pathways is likely to perturb the other networks. The evidence of alteration in glucose utilization during different clinical conditions is plentiful [28,29,30,31,32]. In malaria, several aspects of glucose uptake and metabolism need to be considered. Among these, the most important is the enhanced glucose utilization by the parasite [14]. In this situation, the utilization of glucose by the host tissues has received scant attention, especially during the early stage of the disease. Nonetheless, this aspect is important in understanding the physiological events that prepare the host as the disease progresses. Therefore, in this paper, an attempt has been made to understand the alteration in systemic glucose utilization during the early stage of the disease.

In humans, most *Plasmodium* species result in parasitemia levels limited to a few percent amongst peripheral erythrocytes. Generally, 1% parasitemia is a clinically significant pathological status [14]. In most models of murine malaria, the parasitemia builds up to 20% to 40% or beyond, and in the initial stages of up to about 10% parasitemia no visible pathology is observed [16]. Thus 1% parasitemia is a very early stage for a *P. berghei* malaria model.

It is important to note that while it is inconceivable that parasite metabolism at ~1% of parasitemia could significantly contribute to the metabolic effects seen in the liver and brain, it can still impact the RBC metabolism to some extent, although it is unlikely to be observed under present experimental conditions given the comparatively insensitive nature of NMR spectroscopy. Nevertheless, reconstruction of blood-stage *P. falciparum* metabolic networks indicated the essential nature of several glycolytic enzymes [33]. D-glyceraldehyde-3-phosphate lyase is one such enzyme, that produces glyceraldeyde-3-phosphate from fructose-1,6-biphosphate. Glyceraldehyde-3-phosphate, on the other hand, is a precursor of 2,3-bisphophoglycerate. The data presented here suggest significant alteration in the glucose labeling pattern in the RBCs during the infection, which primarily leads to lower labeling through the 2,3-bisphosphoglycerate shunt of glycolysis. Such an observation was also made earlier in in vitro studies using *P. falciparum* infected RBCs [14]. 2,3-BPG is implicated in oxygen binding to hemoglobin in the RBCs as well as hypoxia [34]. Alteration in this shunt might mean changes in the hemoglobin allostery, the oxygenation status of the cell, as well as alteration in the glycolytic substrate pool. Specifically, hypoxia has been reported in patients with malaria [35]. In fact, decreased activity of 2,3-BPG in parasitized RBCs has been suggested to be a contributory factor to hypoxia [36]. Although we didn’t measure the flux through this pathway, changes in the labeled metabolite pool may implicate altered flux. However, as the parasitemia level in our study is only ~1%, a significant decrease in 2,3-BPG entirely due to parasitized RBCs would mean that the decreased flux through this shunt is not compensated by the majority of the unparasitized RBCs.

The lipidome of RBCs suggests decreased labeling of fatty acids. RBCs do not have an inherent de novo fatty acid synthesis pathway [37]. Instead, fatty acids needed for the house-keeping of the RBC cell membrane are sourced from the liver [38]. Therefore, our observation might mean altered transport of fatty acids. This could also reflect altered lipid homeostasis in general during the infection [38]. As such, the parasite doesn’t have a de novo lipogenesis pathway; instead, it depends upon the host’s fatty acid resources that can be very efficiently scavenged [14]. As such, the requirement of cell membrane-related lipids was also established as an essential metabolic network for *P. falciparum* [33]. It is possible that the altered RBC lipid labeling is a result of elevated demand due to the presence of the parasite at the early stage of the infection. However, without a more targeted method of analysis, the reasons behind altered lipid homeostasis in RBCs will remain speculative.

Significant alteration in the liver glucose utilization was observed during the malarial parasite infection. The data indicate a lowered total pool of metabolites from the branch-out pathways of the glycolysis. Specific observations were made in terms of decreased labeling of C2 of glycerol and cysteine. Instead, elevated labeling pools of glycolysis and TCA cycle metabolites were observed. For example, labeled pools of glutamine, alanine, and lactate were elevated in infected animals. The increased (3-^13^C) glutamine may also mean higher activity of pyruvate through the anaplerotic pyruvate carboxylase (PC) reaction [39], which was reported to occur during ammonia challenge [39] In addition to the PC pathway, a higher level of (2-^13^C) alanine may suggest elevation in alanine aminotransferase activity, suggesting possible hepatocellular injury [40]. Another aspect of this result is the enhanced pyruvate carboxylase activity in the liver of the infected mice since (3-^13^C) glutamine could also be produced via this pathway. PC activity in the liver is implicated in gluconeogenesis. However, this should not be the case here since the mice are under glucose challenge conditions and glycolysis is operative significantly in the infected liver. 

On the other hand, the labeling pattern of lipidome of the liver showed a higher extent of labeling in the unsaturated fatty acid, which may suggest higher desaturase activity. Acetyl-CoA generated from glucose can enter the fatty acid synthesis pathway to generate saturated fatty acids. Fatty acid desaturase then introduces desaturation at a specific position of the carbon chain. The unsaturated olefinic carbons resonate at a downfield region in the NMR spectrum (120–140 ppm, Figure 1) [41]. Although unsaturated fatty acid levels in the RBCs have been implicated in different aspects of malarial infection [42] and their role as antibacterial and immune stimulants has been outlined [43], the specific role of liver desaturase activity is yet to be explored. Further, as mentioned earlier, the transport of fatty acids from the liver may also be perturbed, as seen in the decreased fatty acid levels in RBCs. Therefore, it may be assumed that liver lipid transport and metabolism are collectively altered even at very early stage of the infection.

While analyzing the changes in the glucose utilization pattern in the infected brain compared to uninfected control animals, a distinct pattern was observed during the early stage. The distinction was largely due to the higher extent of labeling in (3-^13^C) glutamate and (2-^13^C) lactate. This indicates an elevated pool of glycolytic metabolites and anaplerotic feed into the TCA cycle. This may also indicate enhanced glutamatergic activity of the infected brain. Changes in the metabolic profile of the brain in the murine model of the disease were implicated earlier [16]. Although the study conducted by Basant et al. [16] cannot directly be compared with the present study as they defined early stage infection when the parasitemia reached ~6%–7% (5th day from inoculation) and did not subject the animals to a labeled glucose challenge, some similarities are notable. For example, a rise in the lactic acid level observed earlier [16] is corroborated by the present study. This proves that the elevation is due to alteration in the glycolytic pathway. However, this is possibly the first demonstration of the cerebral metabolic perturbation (specifically, glucose utilization) in a non-cerebral murine model during very early stage infection, i.e., with parasitemia as low as 1%. In the brain, the pyruvate carboxylase (PC) activity is usually localized in the astrocytes, utilizing TCA cycle intermediate α-ketoglutarate to synthesize glutamate and γ-aminobutyric acid [44]. Therefore the elevation of the anaplerotic labeling pool in the brain might mean the change of the glutamate-glutamine cycle and/or changes in the neurotransmitter pool, and may be associated with altered brain physiology. Further biochemical investigation, specifically comprehensive flux analysis, may be performed in order to understand the underlying pathophysiological consequences of the early changes.

To the best of our knowledge, this study is the first that provides organ level insights into the host glucose metabolism at a very early stage murine malaria. The relative insensitivity of NMR spectroscopy is a substantial limitation. Therefore, further information may be gained by adopting a LC-MS based approach. In addition, temporal alteration of glucose utilization over different degrees of parasitemia may provide meaningful insights into disease pathology and treatment regimes.

## 4. Limitations of the Study

This study aimed to investigate the gross tissue level changes in glucose metabolism by NMR spectroscopy during early stages of malarial infection. In order to mimic the human disease, we kept the parasitemia level close to human infection. However, the pathophysiology of rodent malaria may not translate to human pathophysiology at this parasitemia level. Furthermore, it would be good to compare the metabolism between the host and the parasite RBCs. However, with such a low parasitemia, it was extremely difficult to isolate the 1% of infected cells and observe the labeled pools of metabolites in them. We demonstrated that even at such an early stage of the infection, glucose metabolism is affected significantly in the red blood cells, liver, and brain tissues of the host, and that the total labeled pool of metabolites was altered. However, a more comprehensive understanding of the host glucose metabolism remains to be elucidated through flux calculations of the metabolites. To this end, this study could serve as an important pointer for further investigations of specific metabolic pathways in specific organ/cellular systems.

Another important limitation of this study was the lack of absolute quantification. NMR spectroscopy is a highly quantitative technique. However, in this study, we had to resort to 2-dimensional HSQC experiments for several sample types, specifically, RBCs and the brain, due to the low sample amount. It is well acknowledged that absolute quantification from such experiments is logistically challenging. Nevertheless, the comparison across groups still holds true as relative quantification is proportional to the absolute concentration of metabolites.

## 5. Material and Methods

### 5.1. Animal Experiments

The experiments reported here were carried out as per the ethical approval of the Institutional Animal Ethics Committee in TIFR, Mumbai. Sixteen female Balb/c mice, 6–8 weeks of age, were used in these experiments. The mice weighed ~20–22 g, and maintained at 22 °C ± 2 °C and 12 h day-night cycle. They had free access to standard food pellets and water ad libitum. Half of these mice were injected with 3 × 10^5^ RBCs infected with *Plasmodium berghei* ANKA strain, and the other 8 mice were kept as uninfected control animals. The parasitemia were followed microscopically using Giemsa-stained blood smear, prepared from a blood drop collected from the tail vein.

In infected animals, when the parasitemia reached ~1%, in about 2 days, ~0.85 g of (2^13^C) glucose/kg body weight was injected through the tail vein into all of the mice while immobilizing the animals using a mice immobilizer. After ~30 min of each injection, blood was collected retro-orbitally from the mice in microtubes containing 500 μL of solution containing 0.22% EDTA and 0.9% saline. Immediately thereafter, the mice were sacrificed by cervical dislocation; brain and liver were collected by dissection and snap frozen in liquid nitrogen. 

### 5.2. Preparation of RBC, Liver and Brain Extracts

The blood samples were centrifuged at 600× *g* for 5 min. The RBC pellet, about 400 μL of packed volume, was washed with 400 μL 0.9% NaCl solution and then lysed in distilled water (600 μL) by vortexing and keeping the suspension at 4 °C for 15 min. 500 μL of methanol and 400 μL chloroform was added to the RBC lysates, vortexed and centrifuged at 5000× *g* for 15 min. The methanol-water and the CHCl_3_ portions were separated and the solvent was evaporated off by vacuum concentrator. The dried samples were reconstituted in 700 μL D_2_O (the methanol-water fraction) or CDCl_3_ (the chloroform fraction) and were immediately used for NMR spectroscopy.

The liver and brain tissue samples were prepared using methods as reported before [20]. Briefly, intact frozen tissue was weighed, and 4 mL g^−1^ methanol was added to it followed by 0.85 mL g^−1^ water. The suspension was vortexed, and 2 mL g^−1^ chloroform and 2 mL g^−1^ of water was added. This tissue suspension was homogenized using tissue homogenizer (Wheaton, NJ USA), left for 15 min at 4 °C and centrifuged at 1000× *g* for 15 min. The two layers (methanol/water and chloroform) were collected separately. The methanol/water and the CHCl_3_ fraction were dried off using a vacuum concentrator and reconstituted in 700 μL D_2_O and CDCl_3_ (served the purpose of field frequency lock during NMR experiments) respectively. The samples were used for NMR spectroscopy immediately.

### 5.3. NMR Spectroscopy of Organ and RBC Extracts

All NMR spectra were acquired in a 700 MHz (^1^H resonance frequency) Bruker Avance III NMR spectrometer (Karlsruhe, Germany). Samples were analyzed either by 1-dimensional ^13^C NMR spectroscopy or 2-dimensional ^1^H-^13^C HSQC spectroscopy in case the 1-dimensional spectroscopy signal strength was not enough. Specifically, 1-dimensional ^13^C NMR experiments were performed on the hydrophilic fraction of the liver and hydrophobic fraction of the liver and brain. The rest of the samples was subjected to 2-dimensional ^1^H-^13^C HSQC NMR. To acquire the 1-dimensional ^13^C NMR spectra, 10,000 co-added scans were stored as 16k data points with an inter-scan delay of 0.5 s. The spectral window used was 236 ppm. The ^1^H decoupling was achieved by the power gated decoupling technique using the WALTZ16 decoupler sequence. To process the free induction decays (FIDs), an exponential window function was used with a line broadening factor of 5 Hz, followed by the Fourier transformation. The phase and baseline were manually corrected. In order to assign the peaks in the 1-dimensional ^13^C NMR profile as well as for other sample types, 2-dimensional ^1^H-^13^C HSQC spectra were recorded. In the direct dimension, 2k data points were used for 32 transients, 64 such FIDs were acquired over the indirect dimension over the spectral window of 16 and 220 ppm for the direct and indirect dimension, respectively. The ^13^C decoupling was achieved by a GARP sequence during the acquisition in the ^1^H dimension. The inter-scan delay of 1 s was used in this case. To process these data, zero-filling was done to 4k and 8k points in the direct and indirect dimensions, respectively. This was followed by multiplication of the sine-square window function in both directions with additional line broadening factors of 1 and 0.3 Hz along direct and indirect dimensions, respectively. The spectra were Fourier transformed in both dimensions, followed by manual phase and baseline correction.

### 5.4. Multivariate Statistical Analysis

Principal component analysis (PCA) and orthogonal partial least square discriminant analysis (OPLSDA) were performed on the NMR spectra acquired. Initially, a binning procedure was employed on all the spectra using Amix 3.8.4. The binning formalism was different for the 1-dimensional and 2-dimensional spectra. For the 1-dimensional spectra, the visible peaks were integrated and normalized to the total spectral region to generate the working data matrix. The data were mean centered to execute the PCA and OPLSDA analyses.

To bin the 2-dimesional HSQC spectra, only those regions were used where peaks were visible. For brain hydrophilic fraction, the regions were 80–25 ppm (^13^C dimension) and 4.5–0.5 ppm (^1^H dimension). For RBC hydrophilic fraction, 78–57 ppm (^13^C dimension) and 4.55–2.95 ppm (^1^H dimension) were used. For RBC hydrophobic fraction, 60–0.5 ppm (^13^C dimension) and 3.4–0.5 ppm (^1^H dimension) were used. The visible peaks were assigned to region of interests (RoIs) of various widths and processed further.

The binning was followed by integration of the bins, normalization to the total intensity of the working region and unfolding of the 2-dimensional data matrix into a 1-dimensional shape. The data were mean centered, followed by PCA and OPLSDA using Simca-P+12.0.

In both the cases, the data were visualized by conventional score plots. For the 2-D NMR spectra, the loadings were re-folded using Matlab 7.0.1 into 2-dimensional loading and visualized as contour plots.

## Supplementary Materials

The following are available online at https://www.mdpi.com/2218-1989/10/7/277/s1, Table S1: 13C chemical shifts of significant loadings from liver OPLS-DA analysis. The chemical shifts were putatively identified using human metabolome database (hmdb).
