# Supplementary material for: Early Perturbations in Glucose Utilization in Malaria-Infected Murine Erythrocytes, Liver and Brain Observed by Metabolomics
Source: Metabolites. 2020 Jul 7;10(7):277. doi: 10.3390/metabo10070277 (PMC7407383; doi:10.3390/metabo10070277)
Supplement: Supplementary file 1 [file metabolites-10-00277-s001.pdf]

Supplementary table 1: 13C chemical shifts of significant loadings from liver OPLS-DA analysis. The chemical shifts were putatively identified using hmdb.

Sample type	^{13}C ppm	Assignment
Liver hydrophilic extract	73.58	glycerol C2
	70.47	cysteine C2
	27.21	glutamine C3
	69.31	lactate C2
	54.9	alanine C2
Liver hydrophobic extract	128	polyunsaturated fatty acid
